# Fast Localized Calibration for Spatial‐Spectral Excitation Without Fly‐Back Gradients

**DOI:** 10.1002/mrm.70508

**Published:** 2026-07-09

**Authors:** Michael Schär, Sandeep K. Ganji, Robert G. Weiss, Allison G. Hays

**Affiliations:** ^1^ Russell H. Morgan Department of Radiology and Radiological Science, Division of MR Research Johns Hopkins University School of Medicine Baltimore Maryland USA; ^2^ North America Clinical Science, MR R&D, Philips Cambridge Massachusetts USA; ^3^ Department of Radiology Mayo Clinic College of Medicine Rochester Minnesota USA; ^4^ Department of Medicine, Division of Cardiology Johns Hopkins University School of Medicine Baltimore Maryland USA

**Keywords:** coronary angiography, fat suppression, localized calibration, magnetic resonance imaging, RF pulse design, spectral‐spatial excitation

## Abstract

**Purpose:**

Standard spatial‐spectral excitation pulses apply a fly‐back gradient between sub‐pulses, limiting, for example, how thin the slices can be (> 4 mm). Without fly‐back gradients, slices can be as thin as 1.7 mm but require a phase calibration for the sub‐pulses with inverted gradients due to system imperfections. Here we propose and test a fast (< 1 s), localized pre‐scan enabling thin‐slice frequency‐selective excitation.

**Methods:**

The calibration pre‐scan determines the correction phase between even and odd sub‐pulses by measuring the phase difference of two FIDs excited with either the last or second‐to‐last RF sub‐pulse of the spatial‐spectral pulse. Non‐localized and localized versions of the calibration pre‐scan are tested and validated for water‐only excitation in phantoms and the human heart. As an example, breath‐held coronary MR angiography is performed with a spiral multi‐slice sequence employing the proposed pulses with a slice thickness of 1.7 mm.

**Results:**

The proposed calibration pre‐scan takes 11.1 ms per slice, whether it is localized or not. Localized calibration phases are validated with an image‐based calibration map. Slice profiles are visualized for pulses with different time‐bandwidth products. Fat is suppressed to below 20% and 25% of the surrounding water signal in both phantoms and the human heart, respectively. High‐quality coronary angiograms are demonstrated in all test subjects.

**Conclusion:**

Spatial‐spectral pulses without fly‐back gradients can be calibrated quickly (up to 90 slices in 1 s), enabling thinner slices, higher excitation angles, or pulses with sharper slice profiles using waveforms with higher time‐bandwidth products.

## 1 Introduction

Conventional MRI detects signal from both water and fat. Increased electronic shielding of protons in fat compared to water molecules leads to different resonance frequencies. Fat has a proton spectrum with multiple peaks and its dominant methylene peak is shifted by −3.4 ppm compared to water [1], or Δν_wf_ of about 440 Hz at 3 Tesla. In Cartesian imaging, this frequency shift causes the chemical shift artifact along the frequency encoding direction where fat is displaced compared to water leading to either dark or white edges at water‐fat tissue borders [2], an artifact that is well understood and often accepted in clinical images. However, in non‐Cartesian imaging, such as radial, spiral, or Periodically Rotated Overlapping ParallEL Lines with Enhanced Reconstruction (PROPELLER) [3, 4], fat signal is blurred instead of shifted because the frequency encoding direction is rotated for different parts of k‐space [5], requiring suppression of the fat signal.

Fat suppression in MRI can be achieved with a wide variety of methods [6], including “FAT‐SAT” pre‐pulses (CHESS) [7], short inversion time inversion recovery (STIR) [8], DIXON‐type water‐fat separation [9], and spatial‐spectral water‐only excitation [10]. For the pre‐pulse methods CHESS and STIR, fat signal nulling is optimal at one time point after which fat signal recovers with T_1_. When combined with Cartesian acquisitions, center out k‐space ordering is applied to ensure fat signal is nulled when sampling the center of k‐space where signal contrast is defined. However, in radial and spiral acquisitions, the center of k‐space is sampled with every readout, requiring fat suppression throughout, which can be achieved with either DIXON or spatial‐spectral excitation. DIXON generally provides superior fat suppression when encountering B_0_‐field inhomogeneities and can be combined with deblurring in spiral acquisitions [11]. Spiral DIXON has a higher SNR efficiency compared to non‐fat suppressed Cartesian imaging despite requiring the acquisition of two echoes [12]. However, for fast spiral cardiac imaging at 3 T, spatial‐spectral water‐only excitation is the preferred method because it requires only one echo, enabling real‐time imaging [13] and high‐resolution cine or multi‐slice imaging with acquisition times of 10–20 s to allow breath‐holding [14].

**FIGURE 1:**
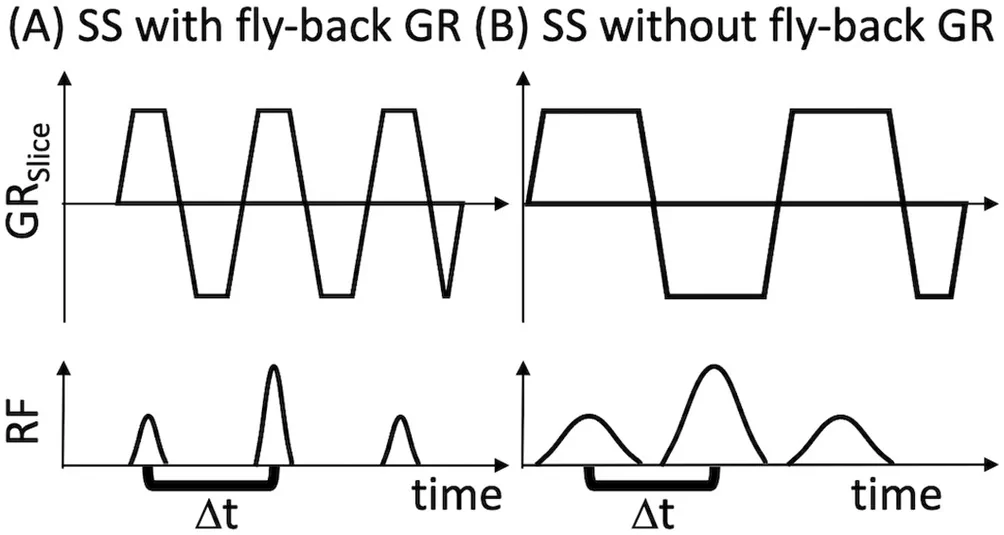
Spatial‐spectral (SS) water‐only excitation pulse with (A) and without (B) fly‐back gradients (GR) showing the radiofrequency amplitude (RF) and slice select GR (GR_Slice_) waveforms. Sub‐pulses need to be separated by Δ*t* = 1.15 ms to generate 180° phase shift between water and fat at 3 Tesla, limiting the size of both GR and RF. Larger GR and RF envelopes in pulses without fly‐back GR (B) allow for thinner slices and larger excitation angles, but system imperfections require a slice location‐dependent phase calibration of the RF pulses applied with inverted GR (2nd RF pulse in (B)) [15].

However, spatial‐spectral pulses are rarely used to excite slices thinner than about 4 mm, and those with higher time‐bandwidth products often have a limited flip angle. Thinner slices are important, for example, for multi‐slice acquisitions that will be reformatted to visualize small structures such as the coronary arteries. On our 3 T scanner the thinnest slice thickness with spatial‐spectral excitation is 7.5 mm, and the flip angle at that slice thickness is limited to 41°. The reason for this and a solution has been presented by Zur [15] in 2000 but was never translated to most vendor systems. In brief, Figure 1 shows example 1‐2‐1 spatial‐spectral pulses using three spatially‐selective sub‐pulses with and without fly‐back gradients. While Zur referred to the pulses with fly‐back gradients as “type i” and to the ones without fly‐back gradients as “type ii,” those pulses are sometimes called “true‐null” or “opposed‐null” [5], respectively. To achieve spectral selectivity, the sub‐pulses have a temporal gap Δ*t* = 1/(2Δν_wf_) = 1.15 ms at 3 T between them so that fat accrues 180° phase while on‐resonant water remains; this will flip fat magnetization back up along the longitudinal axis while water gets excited into the transverse plane. Figure 1A illustrates that fly‐back gradients reduce the sub‐pulse duration and hence the gradient and RF envelopes, and ultimately restrict minimal slice thickness and maximal flip angle as compared to pulses without fly‐back gradients (Figure 1B). Zur explained two important aspects about spatial‐spectral pulses without fly‐back gradients enabling a wide range of applications: (1) An early challenge for water selective pulses without fly‐back gradients was that the inverted gradients under even and odd sub‐pulses lead to opposed phase evolution along the slice direction and, hence, sidelobes with opposite signs that only cancel out and suppress fat signal if the fat along the slice direction is homogenously distributed. This can be overcome by setting the transmit frequency of the pulse to the fat frequency and applying 180° phase jumps between even and odd sub‐pulses. (2) Importantly for this work, spatial‐spectral pulses without fly‐back gradients require a location‐dependent phase calibration between even and odd sub‐pulses that apply positive and negative gradient pulses, respectively, because of unwanted phase shifts between those sub‐pulses due to unavoidable gradient distortions [15]. This calibration requirement is conventionally overcome with fly‐back gradients (Figure 1A), but at the cost of higher minimal slice thickness and reduced maximal flip angles.

Zur proposed a calibration method that determines the phase difference using two spin‐echo acquisitions that apply either a positive or negative sub‐pulse gradient lobe in combination with the off‐center dependent transmit demodulation frequency function during echo acquisition [15]. However, this approach is not straight forward on contemporary scanners because the transmitter and receiver no longer use a physical demodulator. Instead, in this work we propose to determine the phase difference between even and odd sub‐pulses by measuring the phase difference of two FIDs excited with either the last or second‐to‐last RF sub‐pulse of the spatial‐spectral pulse that will be used in the intended acquisition [16]. This simple calibration pre‐scan is fast and can be performed in about 1 s prior to the data acquisition. In an additional validation step, the two calibration scans are combined with imaging readouts to localize the calibration phase, and we show that the optimal calibration phase varies not only from slice to slice but also in‐plane. To localize the signal of the calibration pre‐scan to an organ of interest the recently presented region‐optimized virtual (ROVir) [17] coil combination method can be applied without requiring any other sequence modifications except the need for a coil sensitivity map that is likely already available for other scans in the protocol. Excitation using spatial‐spectral pulses without fly‐back gradients applying fast, localized calibration are demonstrated in both phantoms and the human heart. Lastly, slice profiles at different off‐center locations are shown for pulses with different time‐bandwidth products and both with and without fly‐back gradients.

## 2 Methods

All human studies were approved by the Johns Hopkins School of Medicine Institutional Review Board and informed, written consent was obtained from all study subjects. In vivo studies were conducted on eight healthy subjects with no history of heart disease (30 ± 8 years old, 2 female, mean BMI of 25 kg/m^2^ ranging from 19 to 30). MRI was performed on a 3 T scanner (Achieva, Philips Healthcare, Best, The Netherlands) using a 32‐channel cardiac receive coil and subject‐specific shimming of the excitation radiofrequency field [18].

### 2.1 Implementation of Spatial‐Spectral Pulses Without Fly‐Back Gradient

**FIGURE 2:**
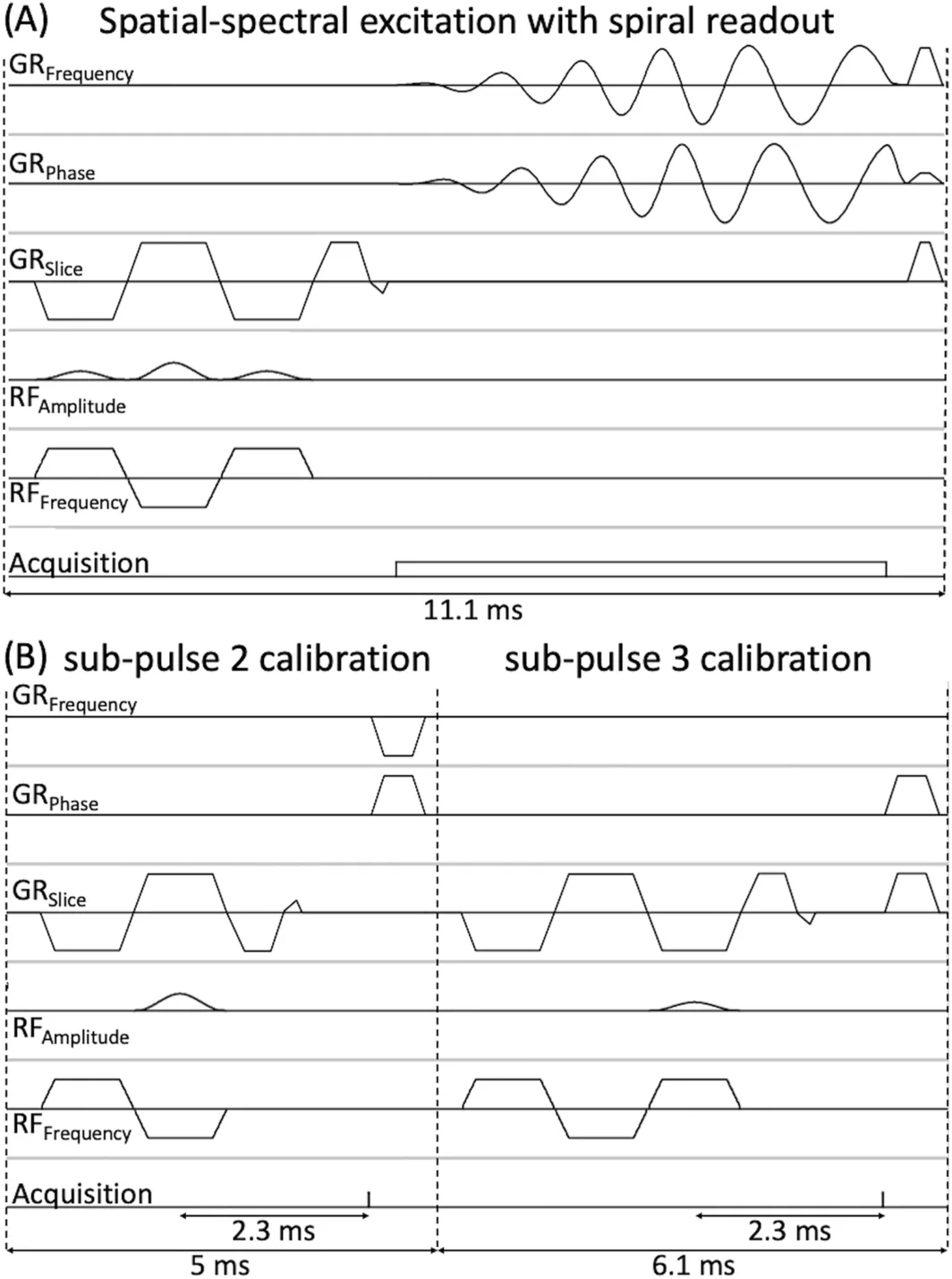
(A) Spatial‐spectral water‐only 90° excitation pulse without fly‐back gradients combined with flow compensated refocusing gradient and a spiral readout as could be used in an efficient spiral multi‐slice acquisition. (B) The two calibration pre‐scans use the same spatial‐spectral excitation pulse but only perform either the last (sub‐pulse 3 calibration) or the 2nd to last sub‐pulse (sub‐pulse 2 calibration). A short FID signal is sampled 2.3 ms after excitation to keep water and fat signal in phase. The phase difference between the two measured signals can then be used as the calibration phase of the second sub‐pulse in (A).

Spatial‐spectral water‐only excitation pulses as provided by the vendor on our scanner use fly‐back gradients and vary the time‐bandwidth product of the RF waveform for either 2D or 3D pulse sequences. The thinnest slice thickness and maximum excitation angle at this slice thickness are listed in Table 1. A research option enables using an RF waveform with a lower time‐bandwidth product enabling 90° excitation, but the slice thickness is still at least 3.9 mm. To achieve thinner slices, spatial‐spectral pulses without fly‐back gradients were implemented. As proposed by Zur [15], the center frequency of the RF pulse is set on fat and 180° phase is applied between sub‐pulses to remove partial volume effects in the fat suppression. To enable longer RF sub‐pulses, RF is also applied during the ramp of the trapezoidal gradient pulses using the variable‐rate selective excitation (VERSE) [19] algorithm to scale the RF waveform accordingly. Flow compensation can be added to the final refocusing gradient if desired. To excite slices at off‐center positions, the RF pulses use a frequency function scaled by the applied gradient waveform. Five different RF waveforms with 0–4 sidelobes were implemented, and their respective time‐bandwidth product, minimal slice thickness, and maximal excitation angle are shown in Table 1. Without applying VERSE to apply RF during the gradient ramps the minimal slice thickness is increased (Table S1). Example RF and gradient waveforms are shown in Figure 2A for a 90° water‐only excitation pulse with a slice thickness of 1.7 mm. Figure S1 shows the RF and gradient waveforms of the standard vendor 2D pulse with fly‐back gradients and in comparison one without fly‐back gradients but a much higher time‐bandwidth product (10 instead of 4.1) that will allow sharper slice profiles for 8‐mm thick slices.

**TABLE 1:** Minimal slice thickness and maximal flip angle (at this slice thickness) for spatial‐spectral pulses with and without fly‐back gradients. All pulses use VERSE to apply RF during the ramp of the trapezoidal gradients. Pulses with fly‐back gradients allow higher flip angles when using thicker slices.

Name	Fly‐back GR	Side lobes	Time*bandwidth	Min. slice thickness [mm]	Max. flip angle [°]
Vendor 2D pulse	Yes	1	4.1	7.5	41
Vendor 3D pulse	Yes	4	10.0	18.3	17
w fly‐back thin	Yes	0	2.1	3.9	90
w/o fly‐back thin	No	0	2.1	1.7	90
w/o fly‐back L1	No	1	4.1	3.2	90
w/o fly‐back L2	No	2	6.0	4.8	69
w/o fly‐back L3	No	3	9.6	7.4	43
w/o fly‐back L4	No	4	10.0	7.8	41

Abbreviations: GR, gradient; L1–L4, 1–4 side lobes; w, with; w/o, without.

### 2.2 Calibration Pre‐Scan to Determine Correction Phase Between Even and Odd Sub‐Pulses

A fast calibration pre‐scan was implemented to measure the phase shift between even and odd sub‐pulses. Figure 2B shows the pre‐scan for the 1‐2‐1 binomial spatial‐spectral water only excitation pulse shown in Figure 2A. The pre‐scan consists of two parts, sub‐pulse 2 and sub‐pulse 3 calibration scans. The sub‐pulse 3 scan excitation is identical to the spatial‐spectral pulse to be calibrated except that only the last RF sub‐pulse is performed enabling to measure the signal phase of the odd sub‐pulses. The phase of the even sub‐pulses is measured in the sub‐pulse 2 scan which only performs the second RF sub‐pulse and has the slice‐select gradient pulse for the last sub‐pulse removed. To refocus the signal, the refocusing and flow compensation gradients are inverted. Both pre‐scans sample a short, 5‐μs FID readout 2.3 ms after the respective excitation to keep water and fat signal in phase. The two pre‐scans are performed consecutively and are repeated for all slices. They last 11.1 ms per slice, allowing phase calibration for 90 slices in under 1 s. This calibration pre‐scan is automatically performed just before the acquisition employing the spatial‐spectral excitation. The complex phase difference between the two signals is determined for all receiver coils and averaged on the spectrometer to be immediately used as the calibration phase for the even RF sub‐pulses, that is, the second RF pulse of the 1‐2‐1 pulse shown in Figure 2. This calibration is referred to as “non‐localized.”

### 2.3 ROVir‐Localized Calibration

Because the image‐based calibration (see below) shows that the calibration phase varies not only from slice to slice but also within each slice, it is desired to determine the optimal calibration phase for the organ of interest. For this, ROVir [17] coil combination is applied. No modifications of the pre‐scan acquisition are needed except that the FID data from receiver coils are first combined using ROVir weights before the complex phase difference is d etermined to localize the calibration phase to a selected area of the body. For this, a coil sensitivity map is needed. Running a quick dummy scan with the same number of slices at the same location triggers the scanner to generate sensitivity maps for the desired geometry. An inline tool (Interactive Data Language IDL version 6.3) was implemented that automatically loads the sensitivity maps and allows the user to either draw a region of interest (ROI) or load the shim volume from the scanner planning interface. ROVir weights are then determined and automatically uploaded to the scanner to be used during the next calibration pre‐scan.

### 2.4 Image‐Based Calibration

To validate the calibration phase determined from the non‐localized and the ROVir‐localized approaches, the two calibration pre‐scans were combined with imaging readouts to map the calibration phase in all imaging slices. The excitation pulses of the sub‐pulse 2 and sub‐pulse 3 calibration scans were used in two multi‐slice acquisitions with the following parameters: acquired/reconstructed FOV = 250/500 mm, acquired/reconstructed voxel size = 3/1.4 mm, 8 spiral segments with a 4.9 ms readout duration, TR/TE = 11.5/2.75 ms, leading to a 16‐heartbeat acquisition. For cardiac scans, ECG triggering and breath‐holding were applied. The complex phase difference in each voxel of the two images determines the local calibration phase. A second inline tool (Interactive Data Language IDL version 6.3) was implemented to calculate the calibration phase maps and to allow the user to manually draw an ROI in each slice. The calibration phases in each ROI were averaged. For acquisitions with image‐based calibration, those calibration phases are automatically uploaded to the scanner and the calibration pre‐scan is skipped.

### 2.5 Phantom Validation

The spatial‐spectral excitation pulses without fly‐back gradient were validated in two identical spherical phantoms (diameter of 100 mm), each half filled with gadolinium doped agar gel (“water,” T_1_ = 150 ms) and baby oil (Well beginnings by Walgreens, “fat,” T_1_ = 213 ms). The two phantoms were placed at iso‐center in *z*‐direction and separated by about 90 mm in right‐to‐left direction covering almost 300 mm in a transversal orientation in order to illustrate the region specific phase calibration. Cartesian multi‐slice data were acquired in an interleaved sequence, acquiring one k‐space line for each slice per TR. The number of slices was chosen to cover the phantoms: 15, 30, and 62 slices for a slice thickness of 7.5, 4, and 1.7 mm, respectively. Other acquisition parameters were FOV 320 × 112 mm^2^, voxel size 1 × 1 mm^2^, TR/TE = 1 s/4 ms, total acquisition time 113 s. 7.5 and 4 mm (research option) thick slices were acquired with the vendor implemented spatial‐spectral pulses using fly‐back gradients and the maximal available excitation angle at this slice thickness of 41° and 90°, respectively. 1.7‐mm thin slices were acquired with the proposed spatial‐spectral pulses without fly‐back gradients and an excitation angle of 90°. Data were acquired with the phase of the 2nd sub‐pulse determined without calibration, non‐localized calibration, ROVir‐calibration, and image‐based calibration. All acquisitions were performed in transversal orientation to have both water and fat in each slice, except one acquisition in coronal orientation where most slices contained either only water or only fat.

### 2.6 Slice Profile Validation

The slice profile of an excitation pulse can be measured with a gradient echo acquisition by rotating the readout gradients along the slice direction. This requires a thin phantom, as the signal in the former readout direction will be projected. A serving tray (diameter 300 mm, height 21 mm) was filled with gadolinium‐doped agar‐gel (T_1_ = 180 ms). Cartesian multi‐slice data were acquired in an interleaved sequence, acquiring one line in k‐space for all slices in each TR. Pulses without fly‐back gradient and a slice thickness of 1.7, 3.3, 4.8, and 8 mm and pulses with fly‐back gradient (slice thickness 4 and 7.5 mm) were tested. The corresponding number of slices was 62, 62, 48, 26, 52, and 28 slices, respectively. Pulse waveform, time‐bandwidth product, and maximal excitation angle were based on Table 1. Other acquisition parameters were FOV = 350 × 300 mm^2^, voxel size = 0.5 × 4 mm^2^ (with the high resolution along the slice profile), TR/TE = 1 s/9 ms, total acquisition time 75 s. Acquisitions were performed in transversal orientation with the center slice at iso‐center. Pulses with a slice thickness ≤ 4 mm were also acquired with 30° rotations around the anterior–posterior axis. The automatic non‐localized calibration was used to determine the phase of the 2nd sub‐pulse for pulses without fly‐back gradients. To test the frequency response, an additional 0.15 mT/m shim gradient was applied along the *y*‐axis. FWHM was measured to determine slice thickness.

### 2.7 In Vivo Validation in the Heart

To validate the fat‐suppression quality of the spatial‐spectral pulse without fly‐back gradients in the heart, axial interleaved Cartesian multi‐slice data were acquired in five subjects. To keep the duration of the expiration breath‐hold to 15 heart beats, a segmented acquisition with a turbo factor of 5 and Compressed Sense factor 3 was applied measuring five lines in k‐space for all slices in each heartbeat triggered to diastole. The acquisition was performed four times with the following calibration methods: non‐calibrated, non‐localized, image‐based, and ROVir‐localized. Other acquisition parameters were: slices = 5, slice thickness = 5 mm, time‐bandwidth product = 6, gap = 8 mm, FOV = 350 mm (right–left, readout) × 280 mm (anterior–posterior), voxel size = 0.75 × 1.3 mm^2^ (with the high resolution along the readout direction), reconstructed voxel size = 0.73 mm^2^, TR/TE = 9.1/4.6 ms.

The fat‐water ratio was determined by selecting a water point whenever the coronary arteries were well visualized and a fat point in the adjacent epicardial fat (see Video S1 for example point placements). Fat‐water ratios acquired with the four different calibration methods were compared against each other using a Friedman multiple comparison test.

### 2.8 Example Application: Coronary Artery MR Angiography

Coronary artery MR angiography (cMRA) was performed in three subjects to demonstrate a potential application for these pulses. Single breath‐hold spiral multi‐slice acquisitions can be used for both an axial scout to plan targeted cMRA scans via the 3‐point plan scan tool [20] and the targeted scans of either the right coronary (RCA) or the left anterior descending (LAD) arteries. Acquisition parameters were: Scout, 30 3.5‐mm thick slices (time*bandwidth = 4.1, non‐localized calibration), FOV = 320 mm, acquired/reconstructed voxel size = 1.2/0.74 mm, TR/TE = 12.4/2.61 ms, 15 spiral segments with a 6.9‐ms readout duration leading to a 15 heart beat acquisition, ECG‐triggered to mid‐diastole; Targeted cMRA, 12 1.7‐mm thick slices (time*bandwidth = 2.1, ROVir‐localized calibration), FOV = 250 mm, acquired/reconstructed voxel size = 0.8/0.5 mm, TR/TE = 14.2/2.7 ms, 19 spiral segments with a 8.2‐ms readout duration leading to a 19 heart beat acquisition, ECG‐trigger delay based on trigger time of corresponding slices in scout. Multiplanar reformatting was performed with the “Soap‐Bubble” tool [21].

## 3 Results

### 3.1 Phantom Validation

**FIGURE 3:**
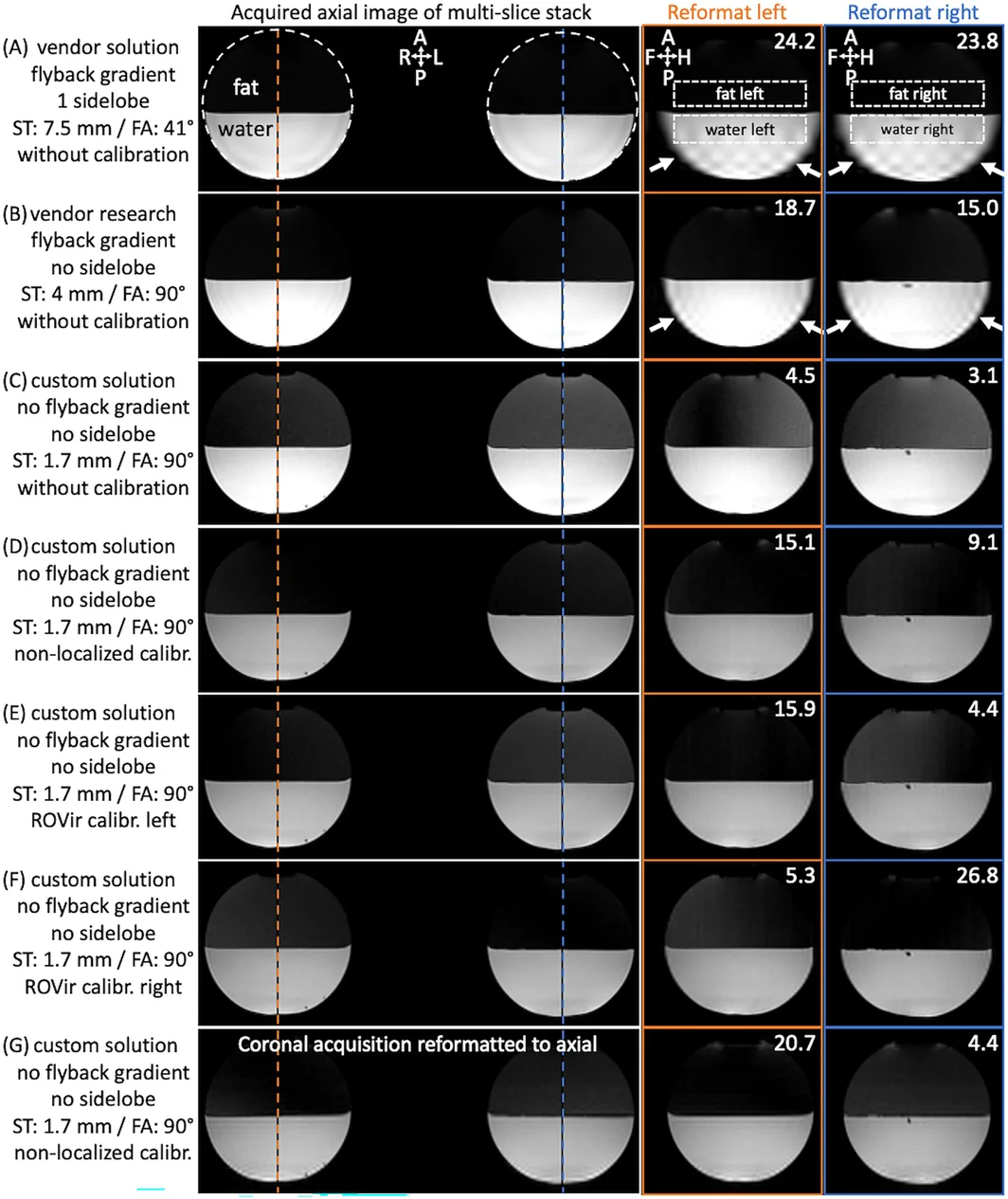
The center slice of an axial multi‐slice stack is shown on the left (A–G), with the corresponding reformats for the left (orange) and right (blue) phantoms on the right. Acquisition details are given on the left including whether fly‐back gradients are used, how many sidelobes in the radiofrequency waveform, slice thickness (ST), excitation flip angle (FA), and calibration method. Locations of the reformatted slices are indicated by the colored dotted line. White dotted lines in (A) show the outline of the spherical phantoms and the areas used to determine the average water and fat signals. The numbers in the top right corner are the water‐to‐fat ratios based on those signals. The white arrows in A and B point to blurred edges in the reformatted images due to thick slices. In (G), the images were acquired in coronal orientation, and both the axial view on the left and the sagittal views on the right are reformats.

Figure 3 shows images of the two water‐fat phantoms acquired simultaneously with spatial‐spectral water‐only excitation pulses. On the left are the center slices in the acquired axial orientation, and on the right are reformatted views along the slices for both phantoms. The top 2 rows (A and B) show images acquired with the vendor provided pulses applying fly‐back gradients. The phantom edges in the reformatted images appear blurred (white arrows) because of the larger slice thickness. The fly‐back gradients enable robust fat‐suppression without the need for calibration. Without fly‐back gradients, the slice thickness can be reduced to 1.7 mm (C–G) leading to sharper edges in the reformatted images on the right. However, if the phase of the 2nd sub‐pulse is not calibrated, the fat suppression quality is reduced and variable (C), as previously shown by Zur [15]. Images in (D) acquired with the automatic non‐localized calibration pre‐scan show consistent fat suppression with a water‐to‐fat ratio of 15.1 and 9.1 in the left and right phantom, respectively. Using ROVir‐based localization to optimize the calibration for the left (E) or right (F) phantom, the water‐to‐fat ratio was improved to 15.9 and 26.8, respectively, at the cost of a reduced ratio in the other phantom. While all slices shown in (A–F) contained both water and fat, (G) shows results from a coronal acquisition where slices contained either only water or only fat to demonstrate that the calibration pre‐scan can handle slices with only water, water and fat, or only fat in them.

**FIGURE 4:**
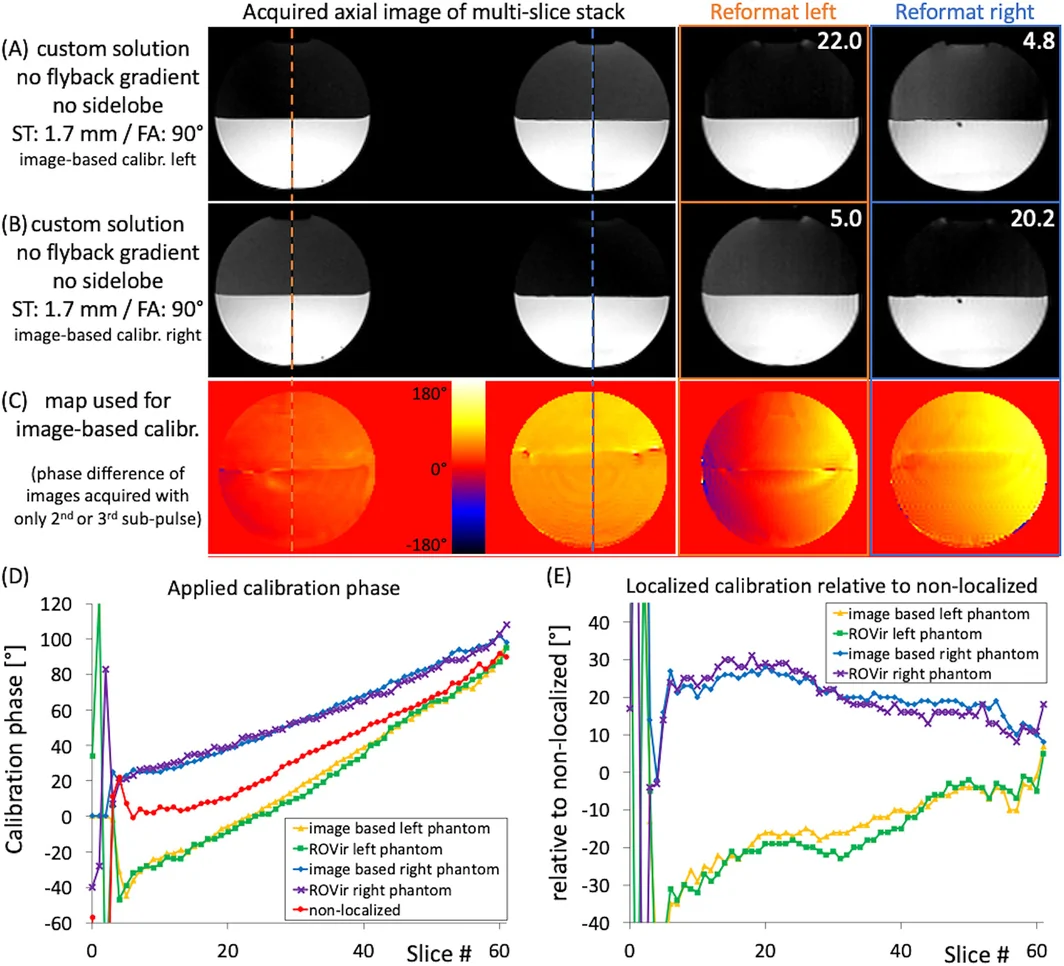
Image‐based calibration to validate ROVir‐based localized calibration. As in Figure 3, (A, B) show the center slice of an axial multi‐slice stack on the left, with the corresponding reformats for the left (orange) and right (blue) phantoms on the right. The images were acquired with the proposed spatial‐spectral pulse without fly‐back gradients and the phase of the 2nd sub‐pulse was set based on the average phase of the left (A) or right (B) phantom in the calibration phase map. (C) Shows the center slice of the calibration phase map on the left, with the corresponding reformats for the left (orange) and right (blue) phantoms on the right. The calibration phase map has consistent values comparing water and fat but shows in‐plane variations of about 20°–50° between the two phantoms depending on the slice. (D) Shows the applied calibration phases and (E) the relative difference of the localized calibration phases relative to the non‐localized calibration in all slices for both ROVir‐based and image‐based calibrations of either the left or the right phantom.

To validate the localization of the ROVir‐localized calibration pre‐scan, a calibration phase map was acquired. The image‐based calibration phase map (Figure 4C) is mostly smooth but has some discontinuities at the water‐fat border due to fat blurring in the applied spiral readout. It shows a calibration offset between the left and right phantom, indicating that different areas may benefit from a locally optimized calibration. Images in Figure 4A,B were acquired with the phase of the 2nd sub‐pulse set based on the average phase in the calibration phase map of the left and right phantoms, respectively, and exhibit robust fat suppression. Figure 4D shows the applied calibration phases for all slices, and that ROVir‐based localization comes to within 10° of the image‐based calibration phase in both the left and the right phantom, validating its localizing ability. Figure 4E shows the localized relative to the non‐localized calibration phase and demonstrates that the optimal calibration phase difference between the two phantoms varies from 20° to 50° depending on the slice. Note that the first and last slices were outside of the phantoms and, therefore, had no signal, leading to erratic calibration phase for those slices.

**FIGURE 5:**
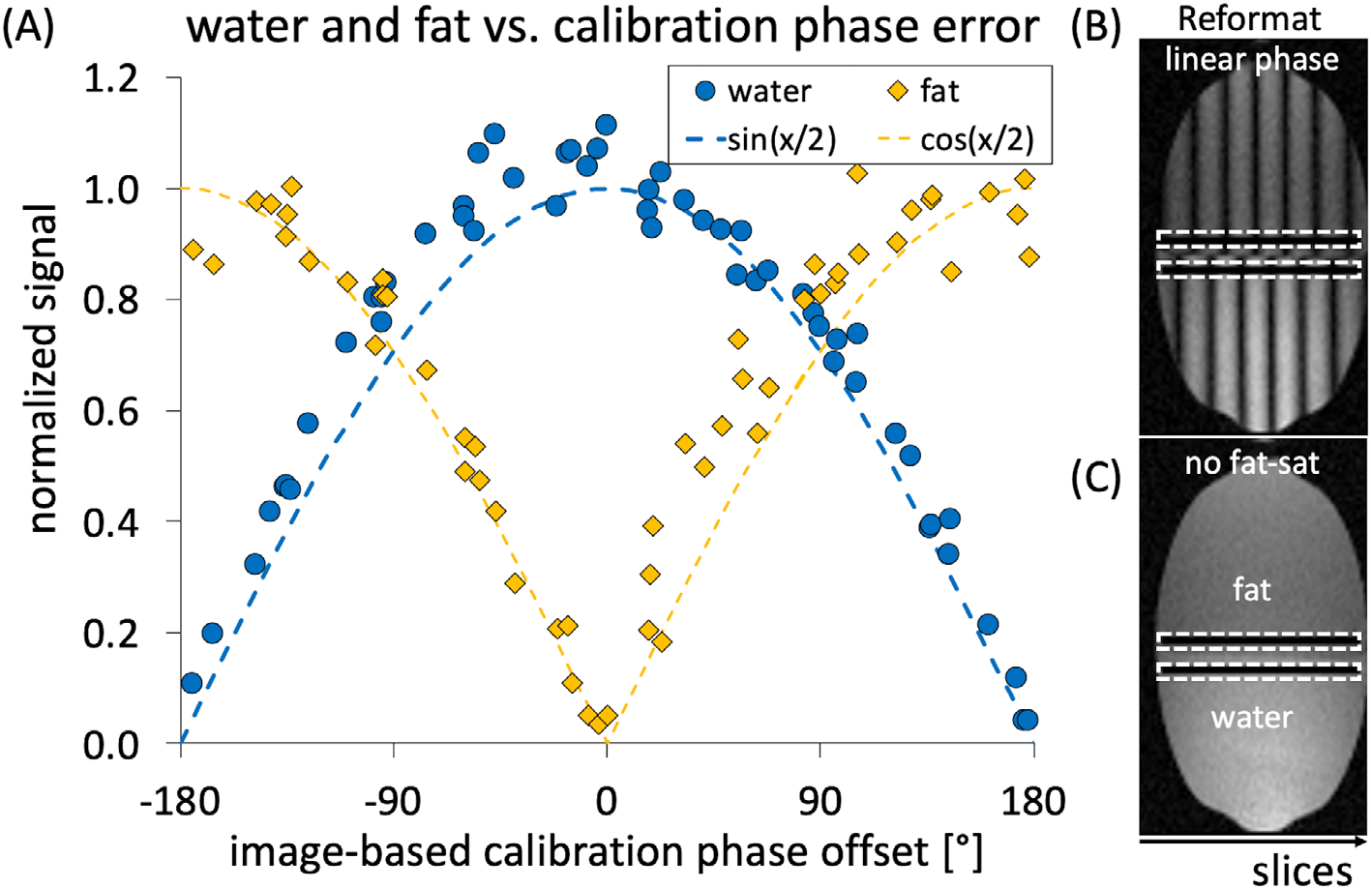
Sensitivity of fat suppression efficiency to errors in calibration phase: (A) Water and fat signals (from the white dotted areas) acquired with an artificial linear phase (B) were normalized with an acquisition without fat suppression (C) and plotted against the offset from the image‐based calibration phase for each respective slice. Water and fat signals approximately follow sin(*x*/2) and cos(*x*/2) functions, respectively.

To test the sensitivity of fat suppression efficiency to errors in the determined calibration phase, Figure 5 plots normalized fat and water signals from an acquisition with an artificial linear calibration phase against the offset from the image‐based calibration phase. The fat signal approximately follows a cos(*x*/2) function, indicating that to suppress fat below 20% the calibration phase needs to be within ±23°.

### 3.2 Slice Profile Validation

**FIGURE 6:**
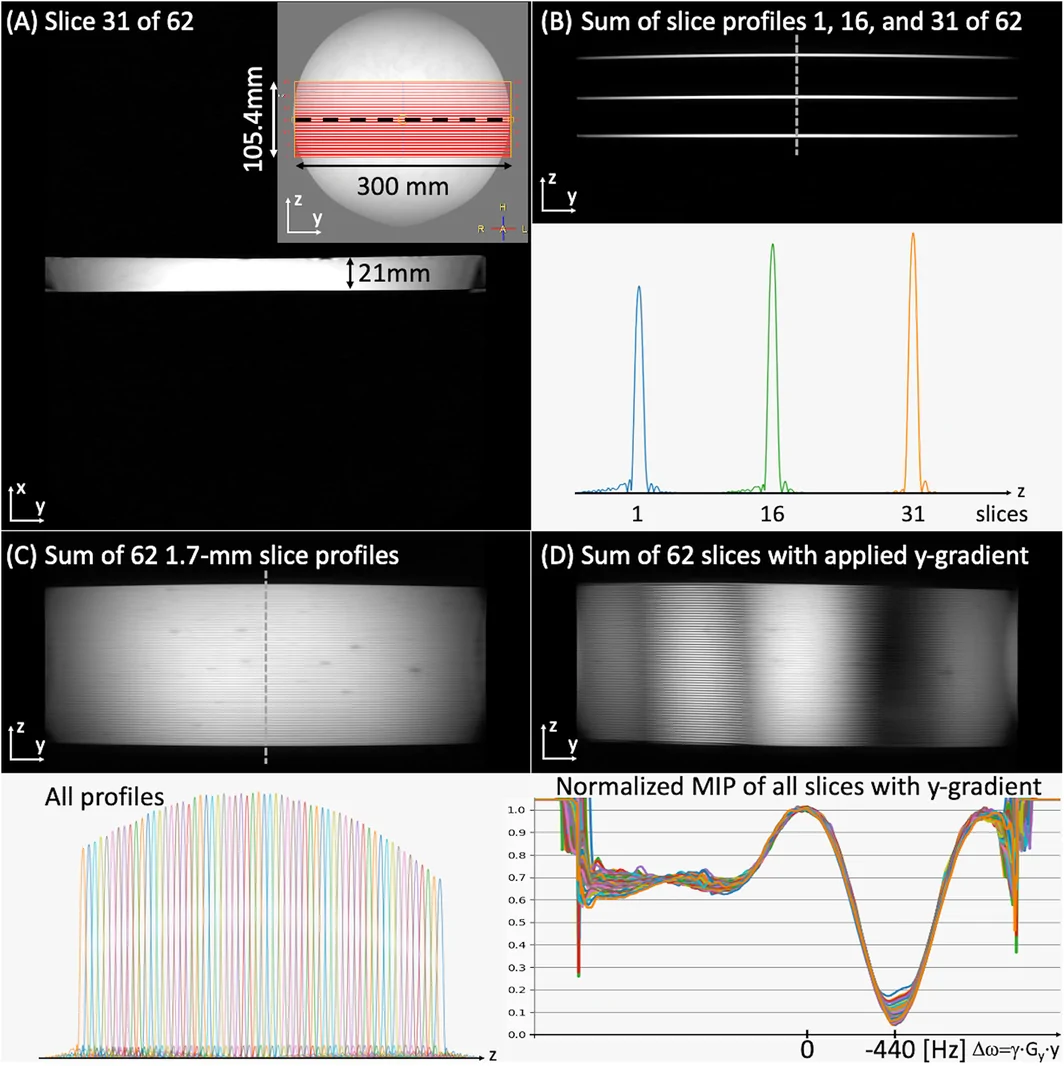
Slice profiles of 1.7‐mm thin slice excited with a spatial‐spectral pulse without fly‐back gradient (time‐bandwidth product 2.1, flip angle 90°) at different off‐center positions. (A) Shows the center slice (black dotted line) of a 62‐slice acquisition in a flat disk‐shaped phantom (planning shown in inset). Rotating the readout direction from *x* in (A) to *z* in (B–D) visualizes the slice profile. (B, C) Show the summed slice profiles of 3 and all 62 slices, respectively. In (D) a gradient was applied along *y* to mimic a frequency axis, showing consistent signal suppression at the frequency of the main fat peak at −440 Hz. The bottom of (D) shows a maximum intensity projection (MIP) of all slice profiles in (D) normalized by the ones in (C) demonstrating fat suppression to below 20%.

The measured profiles for the 1.7‐mm thin slice acquired with the proposed spatial‐spectral pulse without fly‐back gradient are shown in Figure 6. Images of the projected slice profile are shown for 3 (B) or all 62 slices (C). Slice profiles through the iso‐center are shown below the images. The slice thickness FWHM was 1.85 ± 0.02 mm (mean ± SD across all slices). Rotating the stack by 30° around the *x*‐axis (not shown in Figure) led to a FWHM of 1.84 ± 0.02 mm. A *y*‐gradient was applied and shows consistent suppression to below 20% at the fat frequency for all slices (Figure 6D). Figures S2–S6 show the profiles for pulses without fly‐back gradients and higher time‐bandwidth products, and for the vendor provided pulses using fly‐back gradients.

### 3.3 In Vivo Validation in the Heart

**FIGURE 7:**
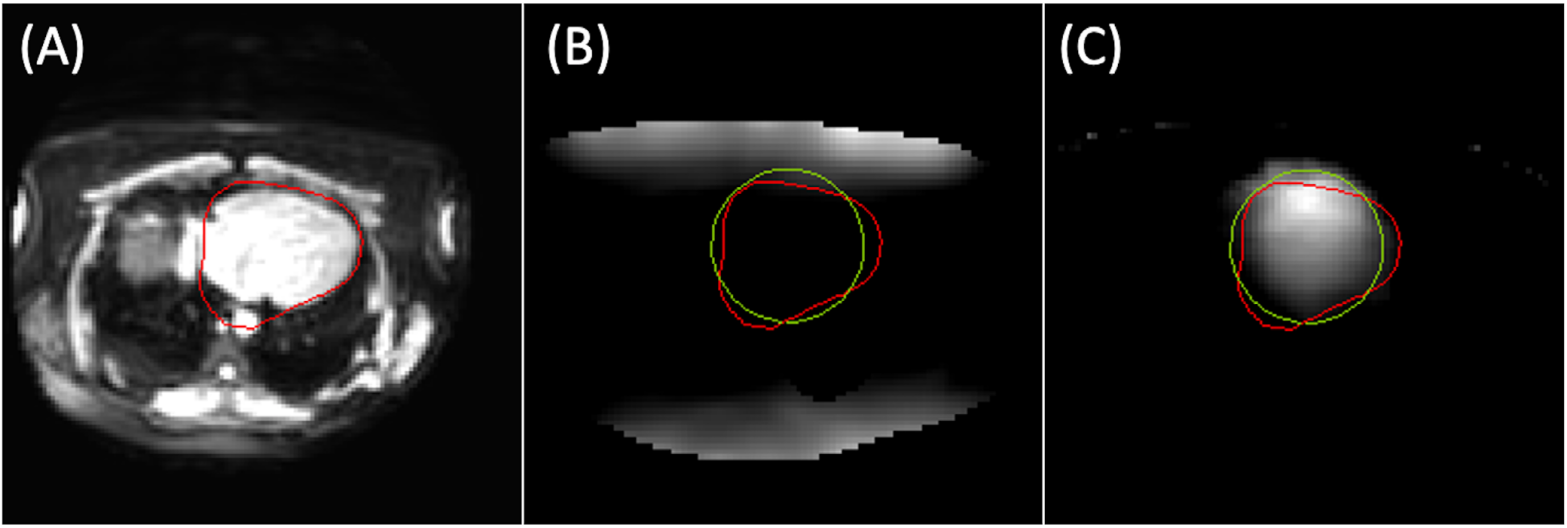
To determine the ROVir weights, a quick dummy scout scan (A) with identical slice locations is performed to trigger the scanner to generate coil sensitivity maps. Summing the coil sensitivity maps with equal weights (B) leads to high signal from locations close to the receiver coils. When combining the sensitivity maps with the determined ROVir weights, only signal from the heart remains (C), enabling localization of the calibration signal. Red line: User defined ROI to select the heart. Green circle: delineating the inner border of the ROVir interference region.

ROVir weights to localize the heart were successfully determined using the inline tool in all subjects. Figure 7 illustrates how the ROVir coil combination is able to localize the signal to the heart.

**FIGURE 8:**
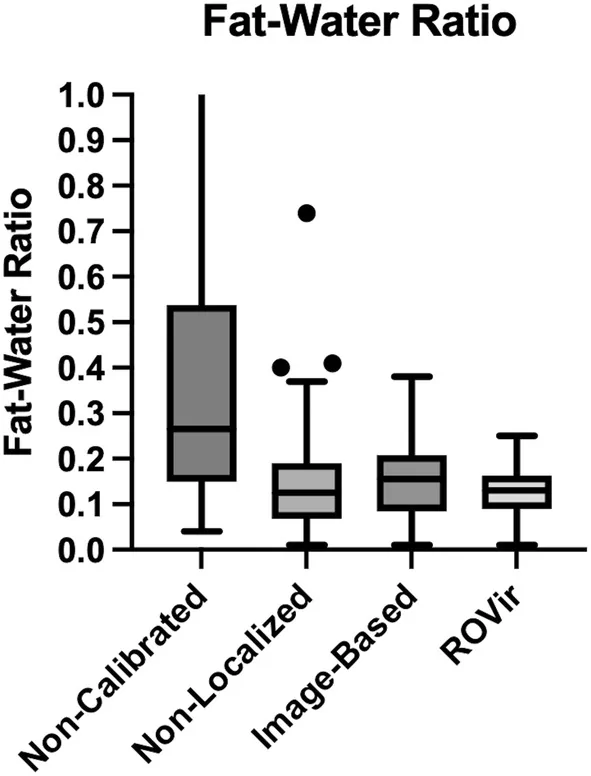
The ratio of epicardial fat signal to coronary artery signal in axial acquisitions using the proposed spatial‐spectral pulse without fly‐back gradients. Shown are Tukey box plots based on 38 measures in five subjects for four different acquisitions. The applied spatial‐spectral pulses were either not calibrated (Non‐Calibrated), or calibrated using the non‐localized, the image‐based localization, or the ROVir‐based localization method.

Figure 8 shows the fat suppression quality around the coronary arteries when using water only excitation with spatial‐spectral pulses without fly‐back gradients. Example images with the selected coronary artery and fat locations in one subject are shown in Video S1. Without calibration the fat suppression is very variable. With calibration, the median fat‐water ratio was 15% or less, and the 75th percentile was 20% or less. Using non‐localized calibration led to a few locations with fat‐to‐water ratios of 40% or more. ROVir‐localized calibration offered the most robust fat suppression with a narrow range and maximal fat‐to‐water ratio of 25%. Friedman multiple comparison test showed that fat suppression using calibrated acquisitions was significantly better than without calibration (all *p* < 0.01), but there was no statistically significant difference between localized and non‐localized calibrations.

### 3.4 Example Application: Coronary Artery MR Angiography

**FIGURE 9:**
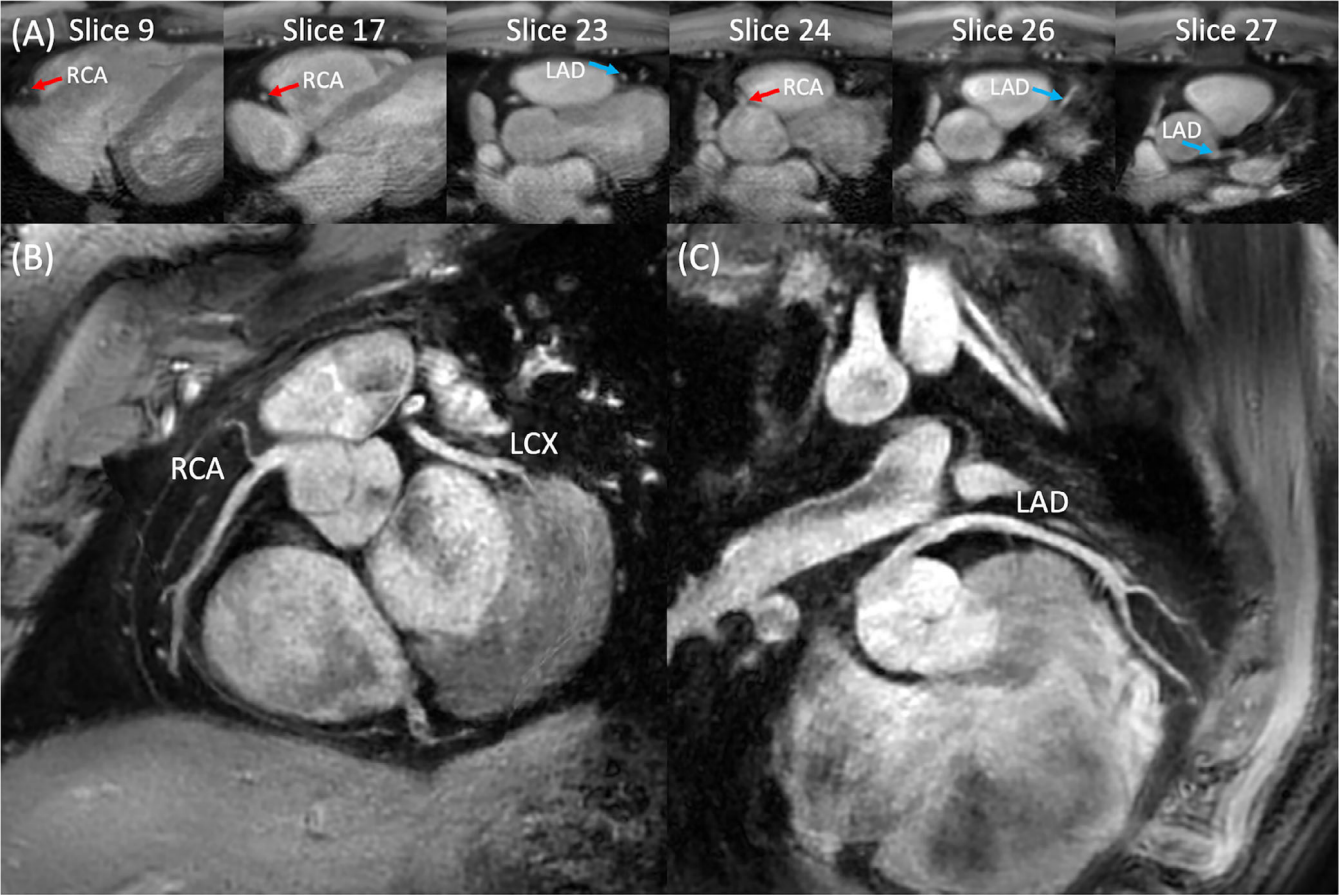
(A) Shows 6 of 30 cropped slices of an axial scout used to plan targeted cMRA of the RCA and LAD. Red and blue arrows indicate the three locations used to plan the cMRAs using the three‐point plan scan tool. Reformats along the RCA (B) and LAD (C) of the targeted multi‐slice acquisitions show high quality angiograms with excellent fat suppression. LAD, left anterior descending; LCX, left circumflex; RCA, right coronary artery.

Figure 9 demonstrates cMRA as an application for the proposed pulses. Both scout and targeted RCA and LAD cMRA with sub‐millimeter in‐plane resolution were acquired in a breath‐hold each. High‐quality cMRA was acquired in all three subjects (Figure S7).

## 4 Discussion

Spatial‐spectral excitation pulses without fly‐back gradients can be calibrated with a sub‐second pre‐scan and enable slice thickness as thin as 1.7 mm or pulses with higher time‐bandwidth products compared to those using fly‐back gradients. Fat signal was suppressed to below 20% and 25% of the neighboring water signal in both phantoms and the human heart, respectively. As a potential application, high‐quality breath‐held coronary angiograms with sub‐millimeter in‐plane resolution were demonstrated.

Image‐based calibration maps, implemented to validate the non‐localized calibration, showed that the calibration phase varied in‐plane and not only from slice to slice as originally expected. Therefore, ROVir [17] coil combination was added to the otherwise identical calibration pre‐scan to locally optimize the calibration for an organ or area of interest. ROVir‐localized calibration was validated against image‐based calibration maps in phantoms, and localized calibration improved fat suppression in the phantoms. In axial acquisitions of the human heart, ROVir‐localized calibration offered the most robust fat suppression as the measured fat‐to‐water ratio had the lowest and most narrow range. However, despite fat‐to‐water ratios of more than 40% in three locations, the non‐localized calibration was not statistically significantly worse than the localized calibrations, maybe because the calibration phase variation across axial slices averages out to the one of the heart close to the center of the body. Nevertheless, we expect that ROVir‐localized calibrations will be more robust especially for oblique scanning in the body. ROVir coil combination has been developed and used to accelerate the image acquisition by suppressing signal from “interference” areas, allowing to reduce the FOV and hence requiring less data in k‐space at the cost of SNR in the signal area of interest [17, 22, 23, 24]. Here we demonstrated that ROVir can also be used to localize pre‐scan/calibration data acquisitions.

The determined calibration phase shown in Figure 4D has a linear component, which could be caused by not perfectly accounting for phase caused by the offset frequency required to excite off‐center slices. To demonstrate that the calibration suggested by Zur [15] is indeed needed, two multi‐slice stacks with identical settings were acquired in both transverse and sagittal orientations: the corresponding calibration phases had an offset of about 30° at iso‐center and a different slope for varying off‐center positions (Figure S8), confirming the need for a slice orientation dependent calibration.

In the current implementation, a quick dummy scan is needed to trigger the scanner to generate sensitivity maps and an inline tool that uses those maps to generate the ROVir weights taking 1–2 min of valuable scanner time. Future work includes automating this step in the scanner's framework using the shim volume from the user interface of the scanner for localization.

Slice profiles of the proposed spatial‐spectral pulse without fly‐back gradients for a range of time‐bandwidth products, and for the vendor provided pulses using fly‐back gradients are shown over a large range of off‐center positions and rotation (Figures 6 and S2–S6). The FWHM of the thin slices was 9% and 6% larger than the nominal thickness, 1.85 and 3.49 mm instead of 1.7 and 3.3 mm, respectively. The pulses with higher time‐bandwidth products, made possible by the lack of fly‐back gradients, had much sharper slice profiles and the mean FWHM of 4.84 and 7.94 mm was within 1% of the nominal 4.8 and 8 mm, respectively. In contrast, the slice profiles from the vendor provided pulses using fly‐back gradients were less sharp for similarly thick slices (due to the lower time‐bandwidth product that can be applied), and increasingly misshapen with stronger sidebands the further off‐center they were.

The proposed spatial‐spectral pulses without fly‐back gradients in this work are used as water‐only excitation pulses to suppress fat signal. While not demonstrated here, these pulses can also be used to excite fat while suppressing water by setting the center frequency of the pulse to water instead of fat [15]. This is supported by Figure 3G demonstrating that the calibration pre‐scan works in slices that contain water, fat, or both.

## 5 Conclusion

Spatial‐spectral excitation pulses on most MR scanners use fly‐back gradients to circumvent the need to calibrate [15] the phase of the RF pulses applied with inverted gradient lobes. However, fly‐back gradients reduce the available time for RF and gradient pulses, limiting the minimal slice thickness, maximal excitation angle, and time‐bandwidth product of the RF waveforms. Here we presented and tested a simple and fast pre‐scan to calibrate spatial‐spectral pulses without fly‐back gradients. This calibration can be localized to an organ or area of interest using ROVir [17] coil combination to optimize fat suppression in that area. Consistent fat‐suppression was demonstrated in the heart, and 1.7‐mm thin slices were used in a spiral multi‐slice sequence to perform coronary angiography with sub‐millimeter in‐plane resolution in a breath‐hold.

## Funding

The primary funding for this investigation was from the National Institutes of Health (NIH) NIBIB R21EB030626 and the Johns Hopkins Discovery Award 2019.

## Conflicts of Interest

Sandeep K. Ganji is an employee of Philips, the manufacturer of equipment used in this study.

## Supporting information


**Table S1:** Minimal slice thickness and maximal flip angle (at this slice thickness) for spatial spectral pulses with and without fly‐back gradients. Listed are the same pulses as shown in Table 1, but here without using VERSE (and hence RF is only applied during the flat top of the trapezoidal gradients) leading to larger minimal slice thicknesses. The maximal flip angle remains similar when comparing the enlarged minimal slice thickness with the thinner slice of the VERSE pulse. For similarly thick slices, the VERSE pulses would allow either higher flip angles or pulses with lower peak power to reduce power deposition.
**Figure S1:** Example RF and slice select gradient waveforms for spatial‐spectral pulses with (A) and without (B) fly‐back gradients. Shown are (A) the vendor provided 2D pulse (1st row in Table 1) and (B) the proposed pulse without fly‐back gradients and highest time‐bandwidth product (bottom row in Table 1). Both pulses have a minimal slice thickness just below 8 mm and a maximum flip angle of 41° for that slice thickness. The pulse without fly‐back gradients offers much sharper slice profiles (Figure S4), especially at off‐center positions compared to the vendor pulse with fly‐back gradients (Figure S6).
**Figure S2:** Slice profiles of 3.3‐mm thick slices excited with a spatial‐spectral pulse without fly‐back gradient (time‐bandwidth product 4.1, flip angle 90°) at different off‐center positions. (A) Shows the summed slice profiles of all 62 slices (planning shown in inset) and (B) a selection of 3 slices. (C) and (D) plot the slice profiles along the dotted lines in (A) and (B), respectively. The slice thickness FWHM was 3.49 ± 0.06 mm (mean ± SD across all slices). Rotating the stack by 30° around the *x*‐axis (E, D) lead to a FWHM of 3.48 ± 0.07 mm.
**Figure S3:** Slice profiles of 4.8‐mm thick slice excited with a spatial‐spectral pulse without fly‐back gradient (time‐bandwidth product 6.0, flip angle 65°) at different off‐center positions. (A) Shows the summed slice profiles of all 42 slices and (B) a selection of 3 slices. (C) and (D) plot the slice profiles along the dotted lines in (A) and (B), respectively. The slice thickness FWHM was 4.84 ± 0.09 mm (mean ± SD across all slices).
**Figure S4:** Slice profiles of 8‐mm thick slice excited with a spatial‐spectral pulse without fly‐back gradient (time‐bandwidth product 10, flip angle 35°) at different off‐center positions. (A) Shows the summed slice profiles of all 26 slices (planning shown in inset) and (B) a selection of 3 slices. (C) and (D) plot the slice profiles along the dotted lines in (A) and (B), respectively. The slice thickness FWHM was 7.94 ± 0.12 mm (mean ± SD across all slices).
**Figure S5:** Slice profiles of 4‐mm thick slice excited with a vendor provided (research option) spatial‐spectral pulse with fly‐back gradient (time‐bandwidth product 2.1, flip angle 90°) at different off‐center positions. (A) Shows the summed slice profiles of all 52 slices (planning shown in inset) and (E) a selection of 3 slices. (C) and (F) plot the slice profiles along the iso‐center lines in (A) and (E), respectively. The slice thickness FWHM was 4.41 ± 0.29 mm (mean ± SD across all slices). In (B, D) a gradient was applied along *y* to mimic a frequency axis, showing consistent signal suppression at the frequency of the main fat peak at −440 Hz without any calibration needed. (D) Further plots a maximum intensity projection (MIP) of all slice profiles in (B) normalized by the ones in (A) demonstrating fat suppression to below 20%.
**Figure S6:** Slice profiles of 7.5‐mm thick slice excited with the standard vendor provided spatial‐spectral pulse with fly‐back gradient (time‐bandwidth product 4.1, flip angle 40°) at different off‐center positions. (A) Shows the summed slice profiles of all 28 slices (planning shown in inset) and (B) a selection of 3 slices. (C) and (D) plot the slice profiles along the dotted lines in (A) and (B), respectively. The slice thickness FWHM was 6.98 ± 0.50 mm (mean ± SD across all slices).
**Figure S7:** Analogous to Figure 9, (A, D) show 6 of 30 cropped slices of axial scouts used to plan targeted cMRA of the RCA (B, E) and LAD (C, F) of the other two subjects. Red and blue arrows indicate the 3 locations used to plan the targeted cMRAs using the three‐point plan scan tool. LAD, left anterior descending; RCA, right coronary artery.
**Figure S8:** Demonstration that spatial‐spectral pulses without fly‐back gradients require a phase calibration between even and odd sub‐pulses that depend on both slice orientation and location. (A) Shows the orientations of a transverse and a sagittal multi‐slice stack centered on a spherical water/fat phantom located at the iso‐center. Images were acquired with such spatial‐spectral excitation pulses without calibration (B) and with non‐localized calibration (C): On the left is an acquired slice at iso‐center, and on the right a reformat across the 62 slices through the center of the phantom (blue dotted line). The numbers in the top right corner are the water‐to‐fat ratios determined in the white dotted box of the reformatted images indicating excellent fat suppression with the phase calibration. (D) Shows the calibration phase that was applied for the acquisition of the images shown in (C). There is about a 30° phase offset between the two orientations at the iso‐center, and a small difference in the slope for varying off‐center positions.

**Video S1:** To test fat suppression quality, fat‐to‐water ratios were determined by selecting water signal in the coronary arteries (white box) and adjacent pericardial fat (white box with black center). Top row shows images and bottom row shows images with the selected locations. From left to right, images acquired with spatial‐spectral excitation pulses without fly‐back gradients without phase calibration, with non‐localized calibration, with image‐based calibration, and on the right with ROVir‐localized calibration.

## Data Availability

A compiled version of the pulse sequence patch is available on reasonable request to Philips research users.
