# Supplementary material for: Fast Localized Calibration for Spatial‐Spectral Excitation Without Fly‐Back Gradients
Source: Magn Reson Med. 2026 Jul 9;96(5):2253–65. doi: 10.1002/mrm.70508 (PMC13527238; doi:10.1002/mrm.70508)
Supplement: Supplementary file 1 [file MRM-96-2253-s002.pdf]

Supporting Information

Fast Localized Calibration for Spatial-Spectral Excitation without Fly-Back Gradients

Michael Schär[1*], Sandeep K Ganji[2,3], Robert G Weiss[1,3], Allison G Hays[1,3]

[1] Russell H. Morgan Department of Radiology and Radiological Science, Division of MR Research, Johns Hopkins University School of Medicine, Baltimore, MD, USA

[2] North America Clinical Science, MR R&D, Philips, Cambridge, MA, USA.

[3] Department of Radiology, Mayo Clinic College of Medicine, Rochester, MN, USA.

[4] Department of Medicine, Division of Cardiology, Johns Hopkins University School of Medicine, Baltimore, MD, USA

Table S1: *Minimal slice thickness and maximal flip angle (at this slice thickness) for spatial-spectral pulses with and without fly-back gradients. Listed are the same pulses as shown in Table 1, but here* **without** *using VERSE (and hence RF is only applied during the flat top of the trapezoidal gradients) leading to larger minimal slice thicknesses. The maximal flip angle remains similar when comparing the enlarged minimal slice thickness with the thinner slice of the VERSE pulse. For similarly thick slices, the VERSE pulses would allow either higher flip angles or pulses with lower peak power to reduce power deposition.*

name	fly-back GR	side lobes	time * bandwidth	min. slice thickness [mm]	max. flip angle [°]
vendor 2D pulse	yes	1	4.1	11.1	47
vendor 3D pulse	yes	4	10.0	27.3	20
w fly-back thin	yes	0	2.1	5.7	90
w/o fly-back thin	no	0	2.1	2.0	90
w/o fly-back L1	no	1	4.1	4.7	90
w/o fly-back L2	no	2	6.0	8.2	69
w/o fly-back L3	no	3	9.6	9.0	37
w/o fly-back L4	no	4	10.0	9.7	36

Abbreviations: GR: gradient, w: with, w/o: without, L1-L4: 1-4 side lobes.

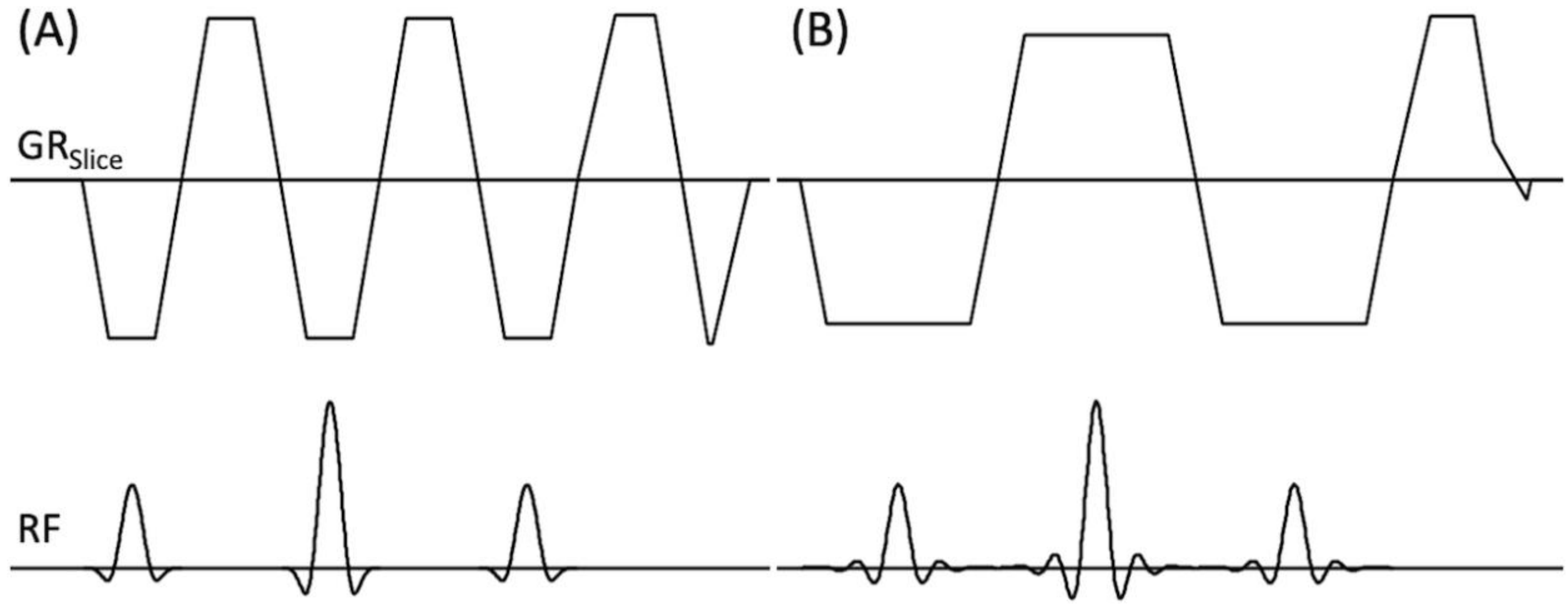

Figure S1: *Example RF and slice select gradient waveforms for spatial-spectral pulses with (A) and without (B) fly-back gradients. Shown are (A) the vendor provided 2D pulse (1st row in Table 1) and (B) the proposed pulse without fly-back gradients and highest time-bandwidth product (bottom row in Table 1). Both pulses have a minimal slice thickness just below 8 mm and a maximum flip angle of 41° for that slice thickness. The pulse without fly-back gradients offers much sharper slice profiles (Figure S4), especially at off-center positions compared to the vendor pulse with fly-back gradients (Figure S6).*

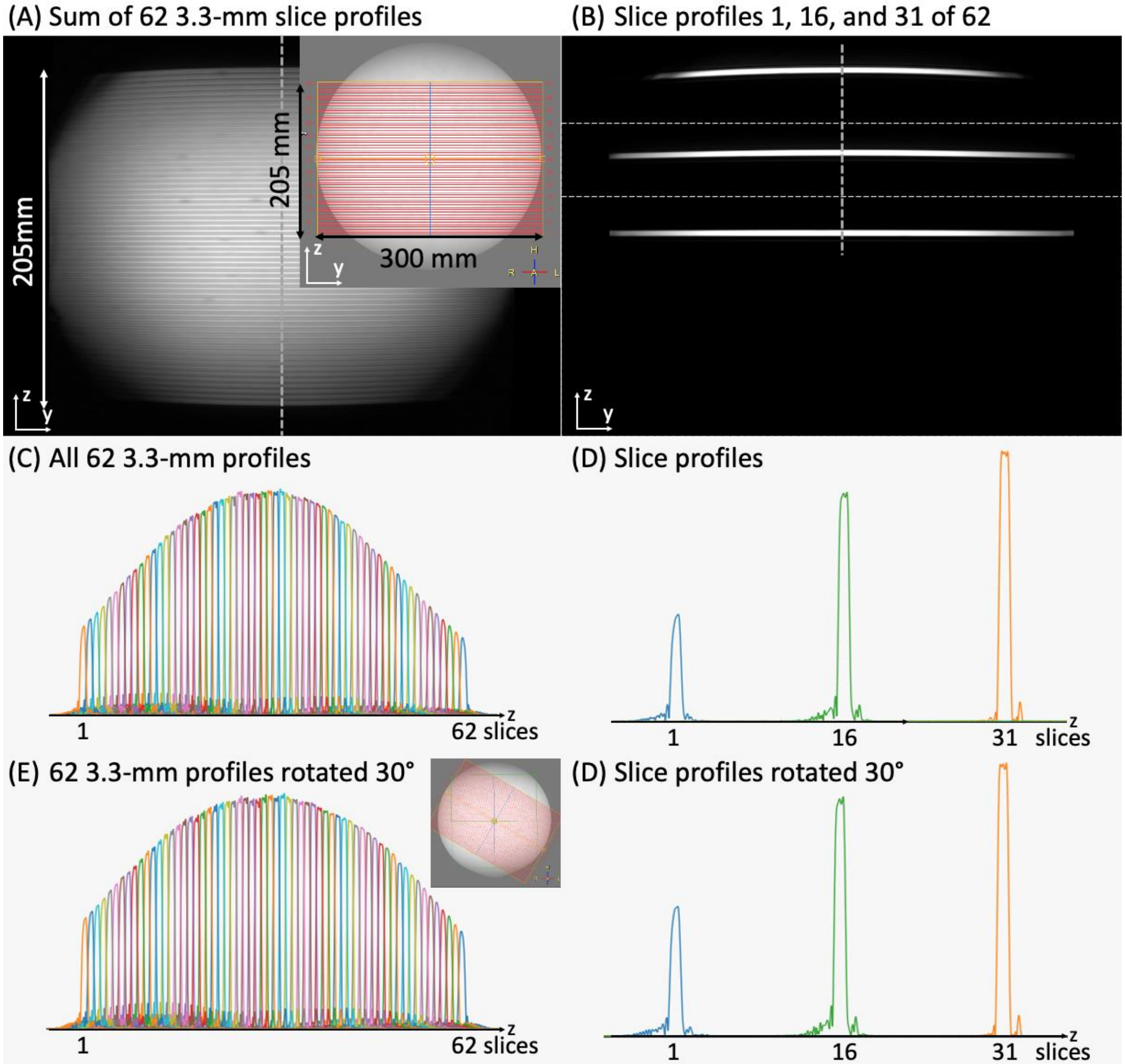

Figure S2: *Slice profiles of 3.3-mm thick slices excited with a spatial-spectral pulse without fly-back gradient (time-bandwidth product 4.1, flip angle 90°) at different off-center positions. (A) shows the summed slice profiles of all 62 slices (planning shown in inset) and (B) a selection of 3 slices. (C) and (D) plot the slice profiles along the dotted lines in (A) and (B), respectively. The slice thickness FWHM was 3.49±0.06 mm (mean±SD across all slices). Rotating the stack by 30° around the x-axis (E, D) lead to a FWHM of 3.48±0.07 mm.*

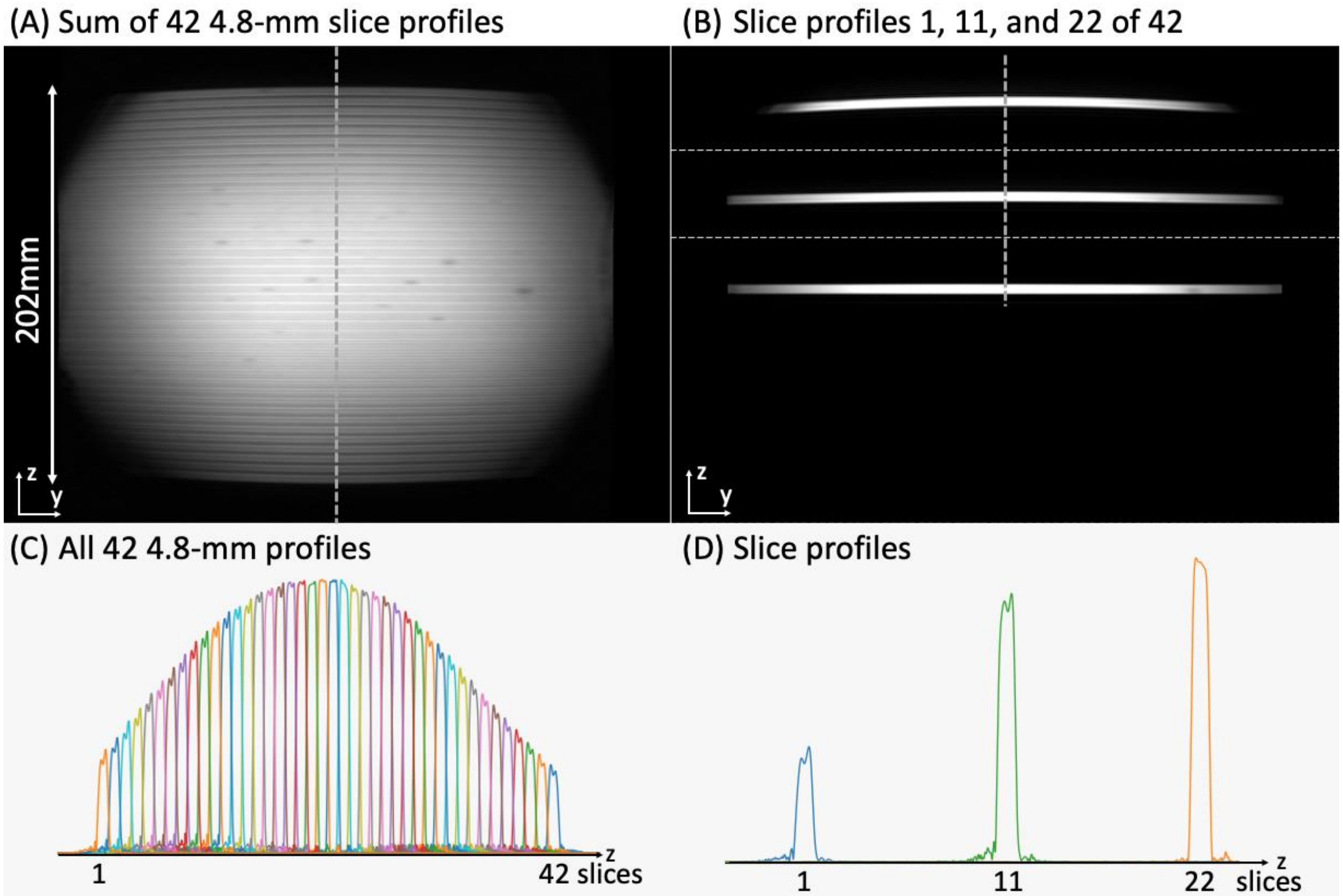

Figure S3: *Slice profiles of 4.8-mm thick slice excited with a spatial-spectral pulse without fly-back gradient (time-bandwidth product 6.0, flip angle 65°) at different off-center positions. (A) shows the summed slice profiles of all 42 slices and (B) a selection of 3 slices. (C) and (D) plot the slice profiles along the dotted lines in (A) and (B), respectively. The slice thickness FWHM was 4.84±0.09 mm (mean±SD across all slices).*

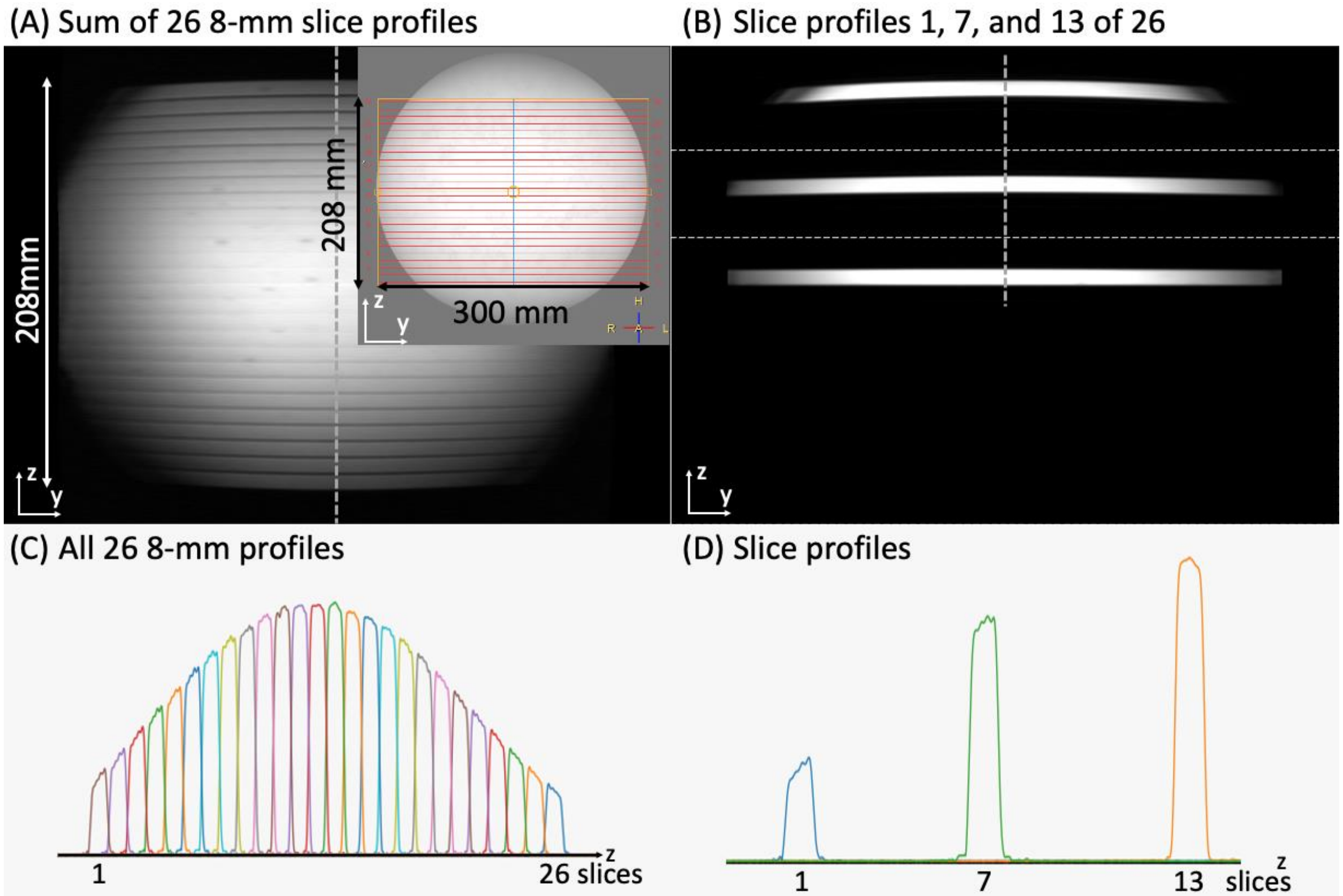

Figure S4: *Slice profiles of 8-mm thick slice excited with a spatial-spectral pulse without fly-back gradient (time-bandwidth product 10, flip angle 35°) at different off-center positions. (A) shows the summed slice profiles of all 26 slices (planning shown in inset) and (B) a selection of 3 slices. (C) and (D) plot the slice profiles along the dotted lines in (A) and (B), respectively. The slice thickness FWHM was 7.94±0.12 mm (mean±SD across all slices).*

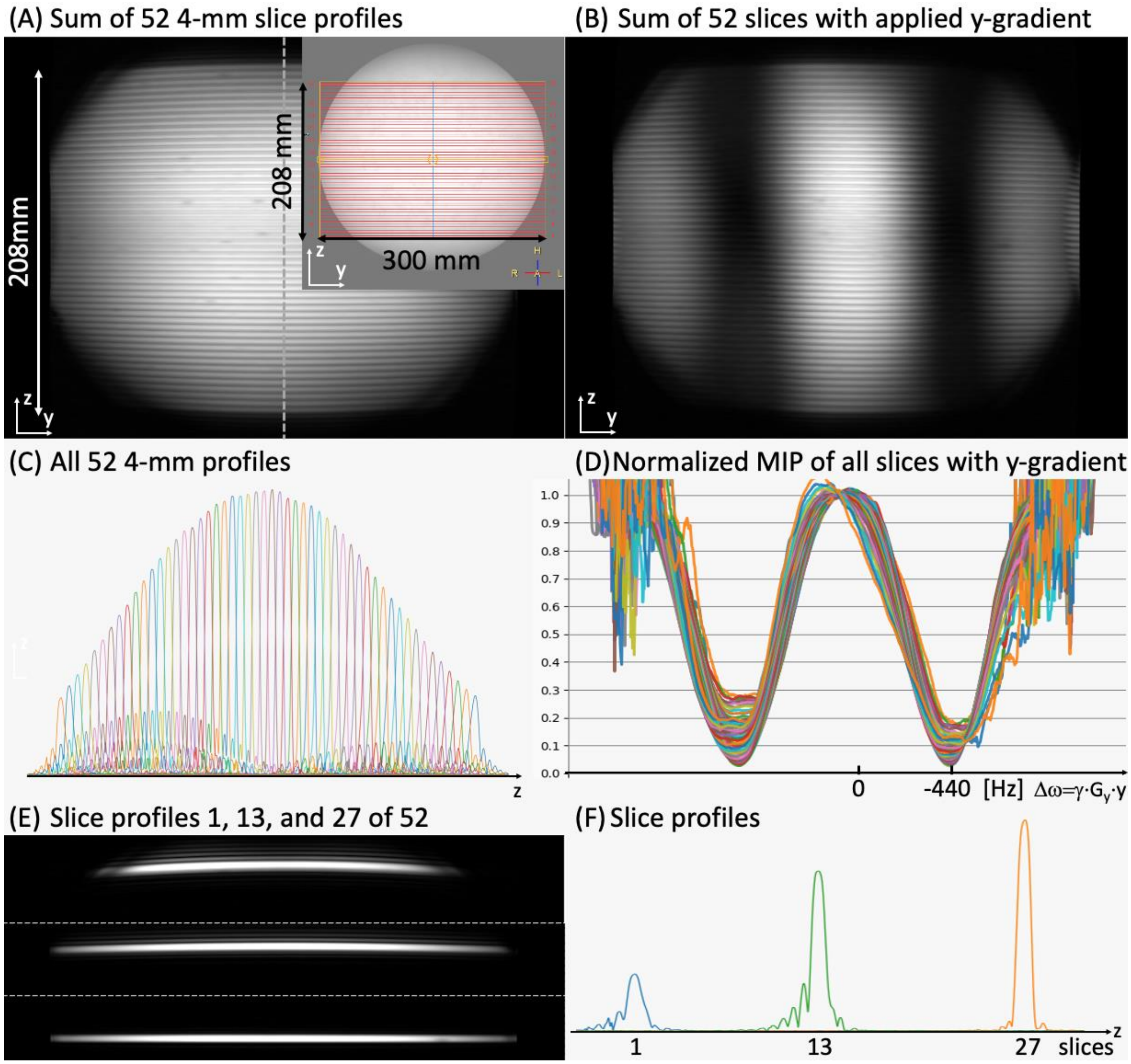

Figure S5: *Slice profiles of 4-mm thick slice excited with a vendor provided (research option) spatial-spectral pulse **with** fly-back gradient (time-bandwidth product 2.1, flip angle 90°) at different off-center positions. (A) shows the summed slice profiles of all 52 slices (planning shown in inset) and (E) a selection of 3 slices. (C) and (F) plot the slice profiles along the iso-center lines in (A) and (E), respectively. The slice thickness FWHM was 4.41±0.29 mm (mean±SD across all slices). In (B, D) a gradient was applied along y to mimic a frequency axis, showing consistent signal suppression at the frequency of the main fat peak at -435Hz without any calibration needed. (D) further plots a maximum intensity projection (MIP) of all slice profiles in (B) normalized by the ones in (A) demonstrating fat suppression to below 20%.*

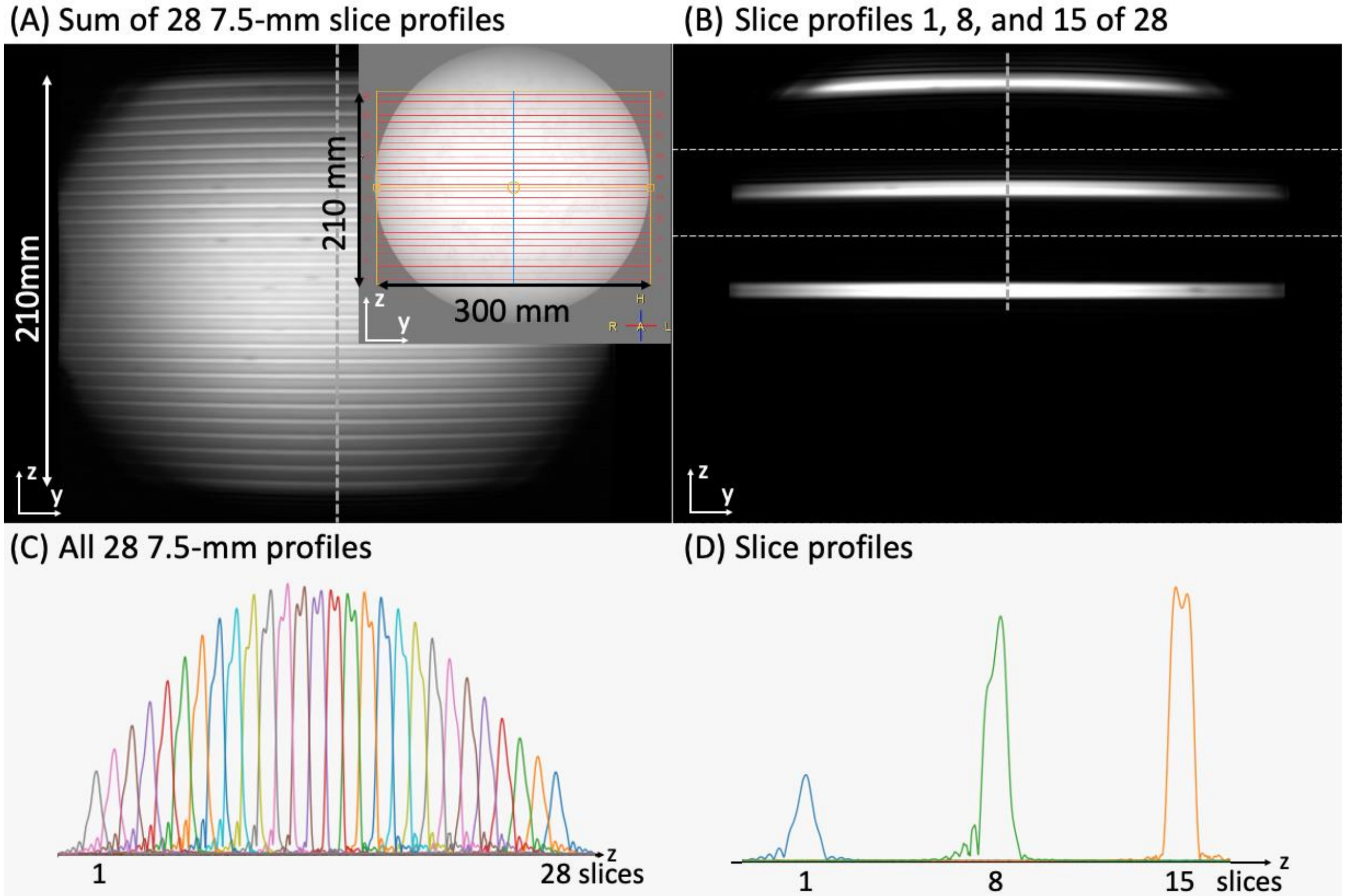

Figure S6: *Slice profiles of 7.5-mm thick slice excited with the standard vendor provided spatial-spectral pulse* ***with*** *fly-back gradient (time-bandwidth product 4.1, flip angle 40°) at different off-center positions. (A) shows the summed slice profiles of all 28 slices (planning shown in inset) and (B) a selection of 3 slices. (C) and (D) plot the slice profiles along the dotted lines in (A) and (B), respectively. The slice thickness FWHM was 6.98±0.50 mm (mean±SD across all slices).*

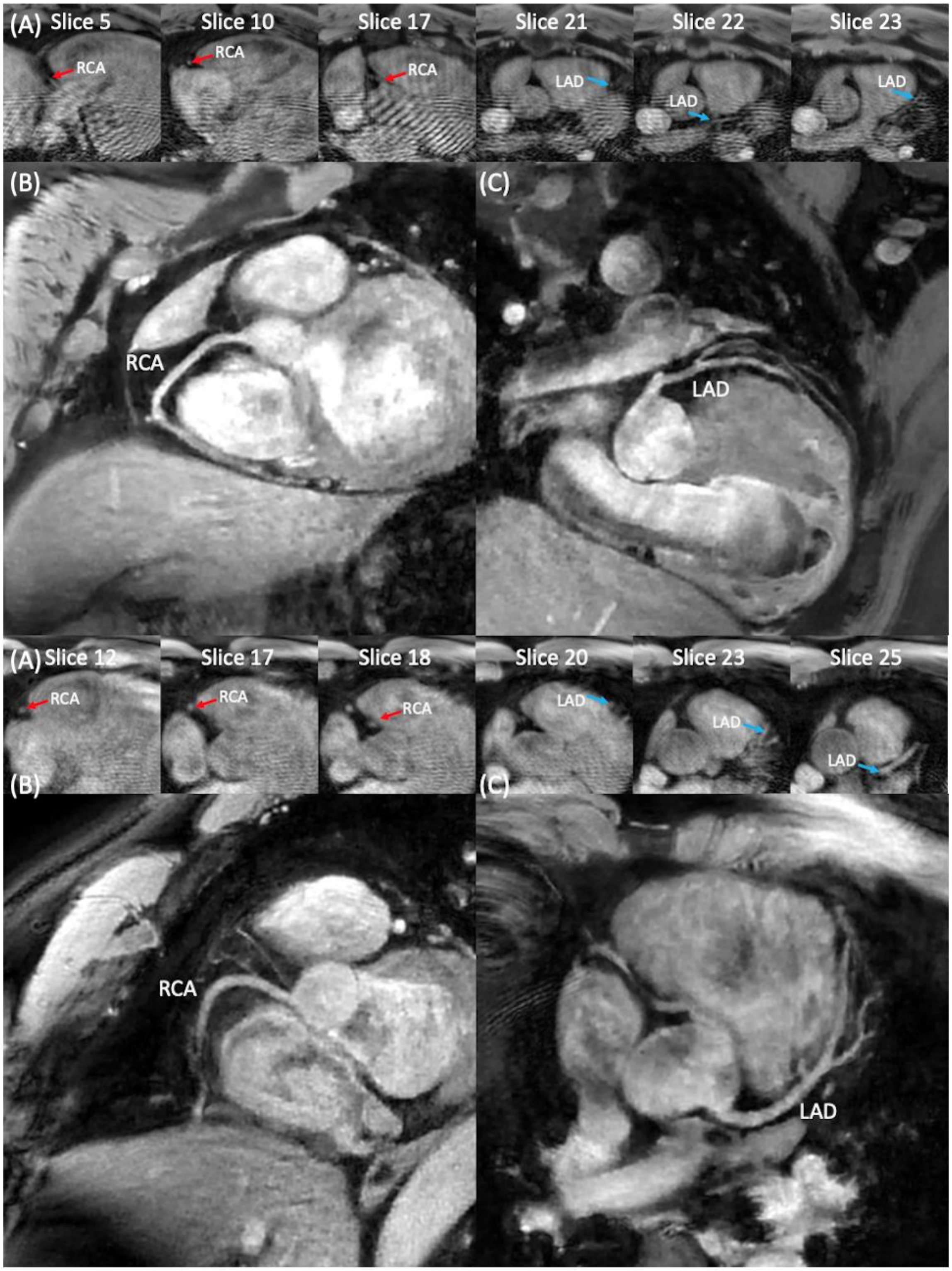

Figure S7: *Analogous to Figure 9, (A, D) show 6 of 30 cropped slices of axial scouts used to plan targeted cMRA of the RCA (B, E) and LAD (C, F) of the other two subjects. Red and blue arrows indicate the 3 locations used to plan the targeted cMRAs using the three-point plan scan tool. Abbreviations: RCA, right coronary artery; LAD, left anterior descending.*

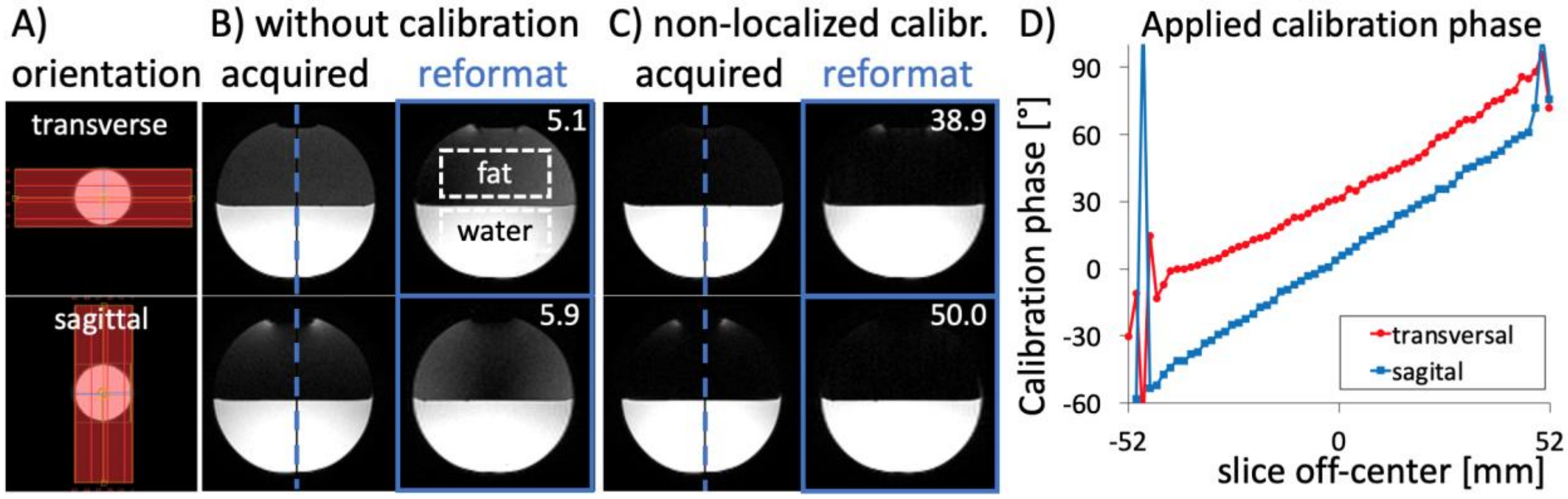

Figure S8: *Demonstration that spatial-spectral pulses without fly-back gradients require a phase calibration between even and odd sub-pulses that depend on both slice orientation and location. A) shows the orientations of a transverse and a sagittal multi-slice stack centered on a spherical water/fat phantom located at the iso-center. Images were acquired with such spatial-spectral excitation pulses without calibration B) and with non-localized calibration C): On the left is an acquired slice at iso-center, and on the right a reformat across the 62 slices through the center of the phantom (blue dotted line). The numbers in the top right corner are the water-to-fat ratios determined in the white dotted box of the reformatted images indicating excellent fat suppression with the phase calibration. D) shows the calibration phase that was applied for the acquisition of the images shown in C). There is about a 30° phase offset between the two orientations at the iso-center, and a small difference in the slope for varying off-center positions.*
